# Machine learning analysis reveals aberrant dynamic changes in amplitude of low-frequency fluctuations among patients with retinal detachment

**DOI:** 10.3389/fnins.2023.1227081

**Published:** 2023-07-20

**Authors:** Yu Ji, Yuan-yuan Wang, Qi Cheng, Wen-wen Fu, Shui-qin Huang, Pei-pei Zhong, Xiao-lin Chen, Ben-liang Shu, Bin Wei, Qin-yi Huang, Xiao-rong Wu

**Affiliations:** ^1^Department of Ophthalmology, The First Affiliated Hospital of Nanchang University, Nanchang, Jiangxi, China; ^2^Department of Radiology, The First Affiliated Hospital of Nanchang University, Nanchang, Jiangxi, China

**Keywords:** retinal detachment, brain region, resting-state functional magnetic resonance imaging, dynamic amplitude of low-frequency fluctuation, sliding window, k-means clustering method, support vector machine

## Abstract

**Background:**

There is increasing evidence that patients with retinal detachment (RD) have aberrant brain activity. However, neuroimaging investigations remain focused on static changes in brain activity among RD patients. There is limited knowledge regarding the characteristics of dynamic brain activity in RD patients.

**Aim:**

This study evaluated changes in dynamic brain activity among RD patients, using a dynamic amplitude of low-frequency fluctuation (dALFF), k-means clustering method and support vector machine (SVM) classification approach.

**Methods:**

We investigated inter-group disparities of dALFF indices under three different time window sizes using resting-state functional magnetic resonance imaging (rs-fMRI) data from 23 RD patients and 24 demographically matched healthy controls (HCs). The k-means clustering method was performed to analyze specific dALFF states and related temporal properties. Additionally, we selected altered dALFF values under three distinct conditions as classification features for distinguishing RD patients from HCs using an SVM classifier.

**Results:**

RD patients exhibited dynamic changes in local intrinsic indicators of brain activity. Compared with HCs, RD patients displayed increased dALFF in the bilateral middle frontal gyrus, left putamen (Putamen_L), left superior occipital gyrus (Occipital_Sup_L), left middle occipital gyrus (Occipital_Mid_L), right calcarine (Calcarine_R), right middle temporal gyrus (Temporal_Mid_R), and right inferior frontal gyrus (Frontal_Inf_Tri_R). Additionally, RD patients showed significantly decreased dALFF values in the right superior parietal gyrus (Parietal_Sup_R) and right paracentral lobule (Paracentral_Lobule_R) [two-tailed, voxel-level *p* < 0.05, Gaussian random field (GRF) correction, cluster-level *p* < 0.05]. For dALFF, we derived 3 or 4 states of ALFF that occurred repeatedly. There were differences in state distribution and state properties between RD and HC groups. The number of transitions between the dALFF states was higher in the RD group than in the HC group. Based on dALFF values in various brain regions, the overall accuracies of SVM classification were 97.87, 100, and 93.62% under three different time windows; area under the curve values were 0.99, 1.00, and 0.95, respectively. No correlation was found between hamilton anxiety (HAMA) scores and regional dALFF.

**Conclusion:**

Our findings offer important insights concerning the neuropathology that underlies RD and provide robust evidence that dALFF, a local indicator of brain activity, may be useful for clinical diagnosis.

## 1. Introduction

Retinal detachment (RD) constitutes the separation of the neurosensory retina from the retinal pigment epithelium. There are multiple types of RD, among which rhegmatogenous is the most common (Steel, 2014). According to a recent report, there are 42 cases of RD per 100,000 people in Germany each year (Gerstenberger et al., 2021). In the early stage of RD, patients often experience acute-onset floaters, flashes of light, and visual field defects (Lumi et al., 2015). When the range of RD invades the macular area, vision decreases to light sensitivity or blindness. Many factors are associated with the occurrence of RD, including high myopia, eye trauma, cataract surgery, a history of retinal tears, and a family history of RD (Verhoekx et al., 2021). Because the retina and optic nerve are regarded as extensions of the central nervous system (CNS), they can be used as windows for assessment of CNS abnormalities (Vujosevic et al., 2023). Accordingly, a link may exist between RD and the CNS.

Currently, the diagnosis of RD mainly relies on optical coherence tomography and B-scan ultrasonography; the results of these examinations can help to identify the type of RD and extent of detachment (Ibrar et al., 2021). However, such examinations only explore ocular visual function in RD patients; it has been unclear whether CNS abnormalities exist in such patients. Recently, the increasing use of resting-state functional magnetic resonance imaging (rs-fMRI) to explore intrinsic brain activity has provided important information concerning the pathological mechanisms involved in RD (Huang et al., 2017; Kang et al., 2019). RD patients reportedly have aberrant functional connectivity (FC) density (Shao et al., 2021) and percent amplitude of fluctuation (Yang et al., 2021) values in various brain regions. Furthermore, Su et al. (2018) discovered that RD patients have altered FC in their default mode network. Thus far, research has mainly focused on changes in the static brain activity of RD patients; there has been a belief that functional interactions among brain regions remain unchanged in time during the whole MRI scan, which is obviously not objective. Chang and Glover (2010) found that when the relationships of the posterior cingulate gyrus were measured over time, FC differed throughout the brain, indicating that relationships among brain regions dynamically fluctuate over time. Since then, there has been increasing evidence that brain activity characteristics exhibit dynamic temporal variation (Sporns, 2011; Abrams et al., 2013). Accordingly, we presumed that analyses of dynamic brain activity would provide insights concerning altered neural mechanisms in RD patients.

The amplitude of low-frequency fluctuations (ALFF) method is useful for measurements of local brain activity. Previous studies have shown that low-frequency oscillations (<0.08 Hz) of blood oxygen level-related (BOLD) signals in the human brain are physiologically significant; such oscillations may represent spontaneous local neural activity (Biswal et al., 1995; Raichle, 2011; Gehrig et al., 2019). Zang et al. (2007) developed the ALFF index and used it to explore the regional intensity of spontaneous fluctuations in BOLD signals. Because the ALFF is calculated under the assumption that the data display temporal stability throughout the acquisition period, it excludes temporal variation in BOLD signals during fMRI scanning. Dynamic ALFF (dALFF) offers a new approach to dynamic brain activity analysis that involves a combination of ALFF and sliding-window methodologies. The dALFF analysis technique has been successfully used to evaluate dynamic changes in brain activity among patients with diabetic retinopathy (Huang et al., 2021), primary dysmenorrhea involving chronic menstrual pain (Gui et al., 2021), and transient ischemic attack (Ma et al., 2021). Additionally, k-means clustering method can cluster the dALFF values of all subjects under different sliding time Windows into several states, so as to better describe the working mode of human brain during the whole scanning time process. Finally, the support-vector machine (SVM) is a supervised machine learning technique that seeks to maximize the margin to sort input points into classes in a high-dimensional space (Pereira et al., 2009). The method of combining SVM and dALFF to analyze changes in brain activity in various diseases has been a research hotspot in recent years, such as comitant exotropia (Chen et al., 2022) and active thyroid-associated ophthalmopathy (Wen et al., 2023) and SVM has high accuracy in distinguishing patients from healthy populations. In the present study, we tested two hypotheses: (1) RD patients exhibit greater temporal variability compared with healthy controls (HCs); and (2) dALFF values are sensitive biomarkers that can distinguish RD patients from HCs.

## 2. Participants and methods

### 2.1. Participants

From January 2023 to April 2023, 23 RD patients and 24 HCs were enrolled in this study. All participants were matched for age and sex; they all were examined in the same clinic and provided written informed consent to participate in the study. All experimental procedures were conducted in accordance with the Declaration of Helsinki, and the study protocol was approved by the Medical Ethics Committee of the First Affiliated Hospital of Nanchang University (Jiangxi Province, China).

The inclusion criteria for RD patients were (1) idiopathic RD involving one or two retinal tears, (2) RD affecting one or two quadrants, and (3) absence of any ocular illness (e.g., cataracts, glaucoma, optic neuritis, or maculopathy) in both eyes. The exclusion criteria for RD patients were (1) recurrent RD or recurrence after RD repair surgery, (2)RD caused by high myopia, (3) ocular trauma-related RD, (4) serious complications associated with RD (e.g., proliferative vitreoretinopathy, vitreous hemorrhage, or macular degeneration), (5) a history of laser treatment or surgery, (6) cardiovascular diseases (e.g., heart disease or hypertension), and (7) psychiatric disorders and cerebral infarction.

According to age, sex, and educational background, HCs were chosen at random from Nanchang City. The inclusion criteria for HCs were the absence of eye diseases and major illnesses (e.g., neurological illness or cerebral infarction); the presence of uncorrected vision or visual acuity better than 1.0; and the completion of MRI-related tests, optical coherence tomography, ultrasonography, and other ophthalmic examinations.

### 2.2. fMRI data acquisition

Rs-fMRI data were collected at the Department of Radiology in the First Affiliated Hospital, Nanchang University, China, using a 3 T MR scanner (Siemens, Erlangen, Germany) equipped with an 8-channel phased-array head coil. The following parameters were used to capture 240 resting-state volumes over 8-min: field of view, 240 mm × 240 mm; repetition time, 2,000 ms; echo time, 40 ms; flip angle, 90°; matrix, 64 × 64; slice thickness, 4 mm; and gap, 1 mm. Thirty axial slices were included in each brain volume. The following three-dimensional MRI parameters were used to acquire high-resolution T1-weighted images of each participant: repetition time, 1,900 ms; echo time, 2.26 ms; flip angle, 9°; field of view, 240 mm × 240 mm; matrix, 256 × 256; number of sagittal slices, 176; and slice thickness, 1 mm.

### 2.3. fMRI data preprocessing

All data preprocessing was conducted using SPM12 and RESTplus (Jia et al., 2019) version 1.25 running in matlab2017b. The data preprocessing steps were as follows: (1) Data collation and classification. (2) Conversion of file format from DICOM to NIFTI. (3) Removal of the first 10 time points. (4) Slice timing correction. (5) Head movement correction. (6) Normalization (standardization of individual space to Montreal Neurological Institute [MNI] standard space). (7) Spatial smoothing. (8) Detrending. (9) Regression of nuisance covariates.

### 2.4. dALFF variance computing

The Time Dynamic Analysis toolbox in RESTplus version 1.25 was used to calculate dynamic metrics. Appropriate window length is essential for dynamic analysis, and sliding windows have key roles in the assessment of dynamic spontaneous brain activity. Previous studies showed that an excessively short window length can increase dALFF signal instability, whereas an excessively long window length does not adequately reflect dynamic temporal changes in dALFF (Leonardi and Van De Ville, 2015; Li et al., 2019). To minimize subjective error caused by a single window length, we used a window length and step size of 1TR for 30TR (60 s), 50TR (100 s), and 80TR (160 s) to calculate the dALFF for each participant. For each participant’s window-based ALFF map, we calculated the mean and standard deviation of each voxel, then determined the appropriate coefficient of variation (CV = standard deviation/mean). Further statistical analyses were conducted using the CV maps.

### 2.5. Statistical analysis

One-sample t-tests were used for statistical analyses of the CVALFF maps of RD patients and HCs. Two-sample *t*-tests were used to assess differences in CVALFF maps between groups. The Gaussian random field (GRF) method was used to correct for multiple comparisons and regressed covariates of age and sex (two-tailed, voxel-level *p* < 0.05; GRF correction, cluster-level *p* < 0.05).

### 2.6. Clustering analysis

To determine the dALFF occurrence state, a k-means method was used to the dALFF values for each participant. The k-means algorithm combines together data that is related in “k” ways, ensuring that the total of squares within clusters is as small as possible (Zhang et al., 2018). The Manhattan (L1) distance function method was performed to assess the reoccurrence over time in patterns of ALFF. All of the dALFF windows were clustered using the clustering centroids for the departure points.

### 2.7. Support vector machine analysis

To determine whether changes in dynamic metrics can be used as diagnostic indicators of RD, we investigated possible diagnostic indices using Gaussian radial basis function kernel SVMs and the LIBSVM software package (Pereira et al., 2009). The steps were as follows: (1) region of interest signal values were extracted from all differential brain regions; (2) a NII file was created to mask differential brain regions; and (3) the radial basis function of the grid search optimization algorithm was used to calculate parameters.

## 3. Results

### 3.1. Demographic characteristics

This study included 23 RD patients (12 men and 11 women; mean age, 51.70 ± 19.37 years) and 24 HCs (11 men and 13 women; mean age, 50.46 ± 14.55 years). Demographic characteristics are shown in Table 1.

**Table 1:** Demographic characteristics of RD patients and HCs.

Characteristic	RD patients	HCs	*p*-value
Men/women	12/11	11/13	0.664^x^
Age (years, mean ± SD)	51.70 ± 19.37	50.46 ± 14.55	0.225^t^
Duration of detachment (days)	15 (7, 90)^a^	N/A	N/A
IOP (mmHg, mean ± SD)	14.52 ± 4.62	N/A	N/A
Axial length of eye (mm, mean ± SD)	24.36 ± 1.88	N/A	N/A
Corneal endothelial cell count (mm^2^, mean ± SD)	2,317 ± 512.24	N/A	N/A
HAMA score	3.89 (2, 5)^a^	N/A	N/A

^x^Data were obtained using Pearson’s Chi-square tests; ^t^Data were obtained using two-sample *t*-tests; ^a^Median (interquartile range); SD, standard deviation; RD, retinal detachment; HCs, healthy controls; IOP, intraocular pressure; HAMA, Hamilton Anxiety Scale; N/A, not applicable.

### 3.2. Differences in dALFF values

**Figure 1:**
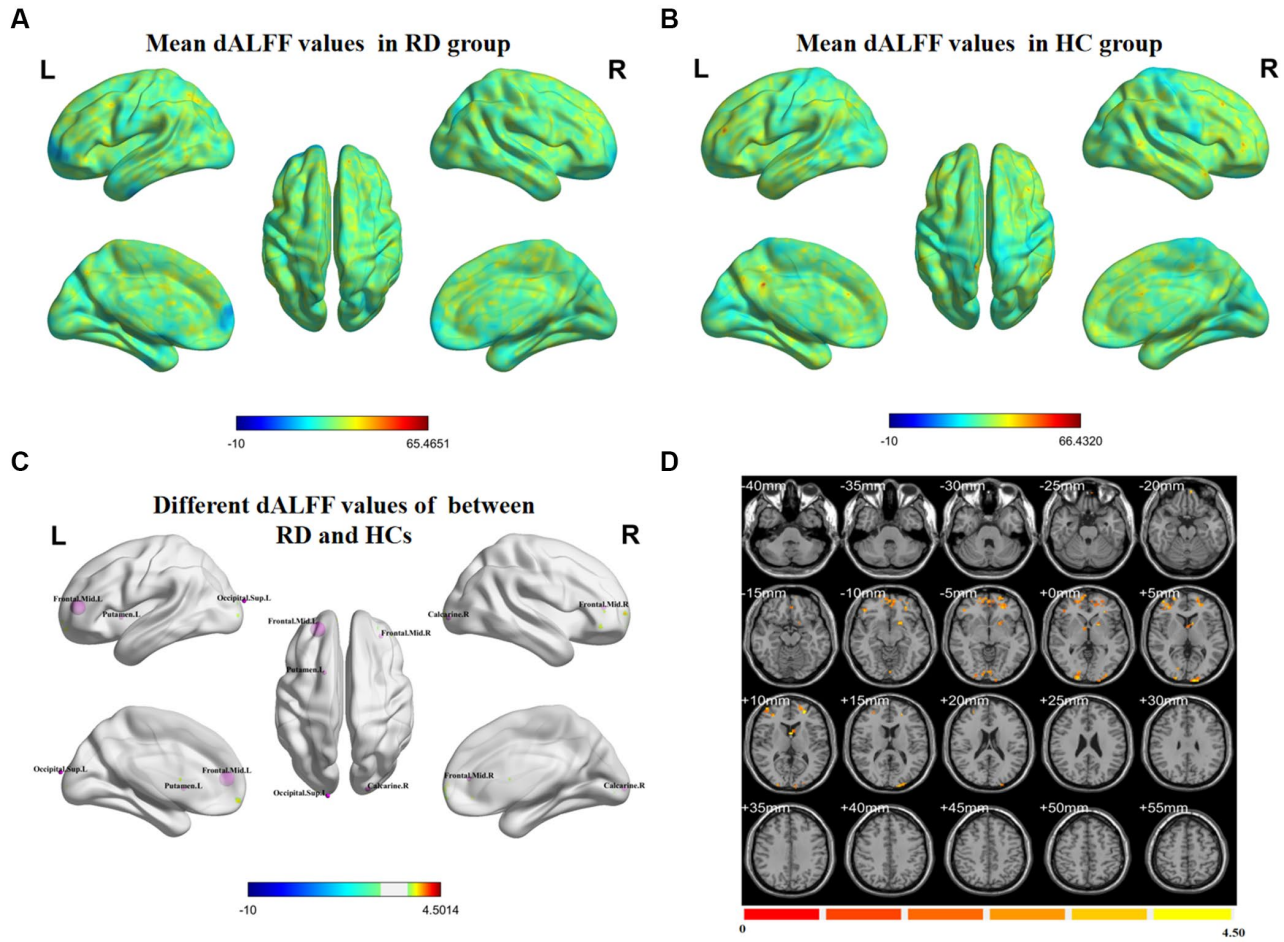
Spatial distributions of dALFF values in **(A)** RD patients (one-sample t-test) and **(B)** HCs (one-sample *t*-test). **(C,D)** Spatial distributions of dALFF values between RD patients and HCs (two-sample *t*-test). HCs, healthy controls; RD, retinal detachment; dALFF, dynamic amplitude of low-frequency fluctuation; L, left; R, right (two-tailed, voxel-level *p* < 0.05; GRF correction, cluster-level *p* < 0.05).

Figure 1 shows the spatial distribution of dALFF values between RD patients and HCs at a sliding window size of 30TR. In the bilateral middle frontal gyrus, left putamen, left superior occipital gyrus, and right calcarine, dALFF values were significantly higher in RD patients than in HCs (Figure 1; Table 2; two-tailed, voxel-level *p* < 0.05; GRF correction, cluster-level *p* < 0.05).

**Table 2:** Significant differences in dALFF values between RD patients and HCs at a sliding window size of 30TR.

Brain region	BA	Peak *t*-score	MNI coordinates (x, y, z)	Cluster size (voxels)
Frontal_Mid_L	–	4.5014	−24, 48, 9	232
Putamen_L	–	4.1322	−18, 9, 0	59
Frontal_Mid_R	–	3.5413	33, 42, 9	63
Occipital_Sup_L	–	4.0016	−15, −102, 15	56
Calcarine_R	–	3.595	21, −96, 0	67

dALFF, dynamic amplitude of low-frequency fluctuation; HCs, healthy controls; RD, retinal detachment; BA, Brodmann area; MNI, Montreal Neurological Institute; Frontal_Mid_L, left middle frontal gyrus; Putamen_L, left putamen; Frontal_Mid_R, right middle frontal gyrus; Occipital_Sup_L, left superior occipital gyrus; Calcarine_R, right calcarine.

**Figure 2:**
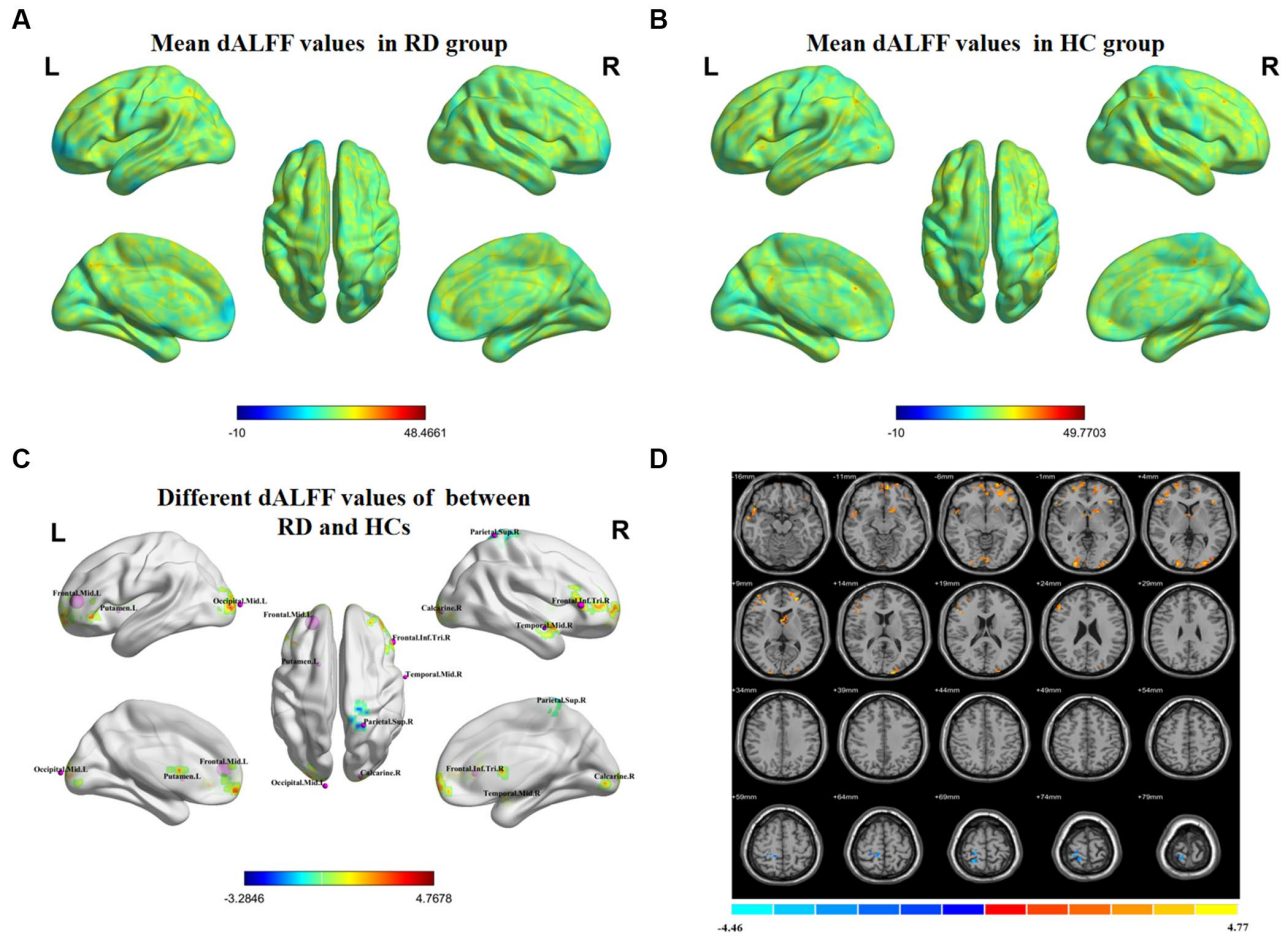
Spatial distributions of dALFF values in **(A)** RD patients (one-sample t-test) and **(B)** HCs (one-sample *t*-test). **(C,D)** Spatial distributions of dALFF values between RD patients and HCs (two-sample *t*-test). HCs, healthy controls; RD, retinal detachment; dALFF, dynamic amplitude of low-frequency fluctuation; L, left; R, right (two-tailed, voxel-level *p* < 0.05; GRF correction, cluster-level *p* < 0.05).

Figure 2 shows the spatial distribution of dALFF values between RD patients and HCs at a sliding window size of 50TR. In the right middle temporal gyrus, left middle frontal gyrus, right calcarine, left putamen, left middle occipital gyrus, and right inferior frontal gyrus, dALFF values were significantly higher in RD patients than in HCs. Conversely, in the right superior parietal gyrus, dALFF values were significantly lower in RD patients than in HCs (Figure 2; Table 3; two-tailed, voxel-level *p* < 0.05; GRF correction, cluster-level *p* < 0.05).

**Table 3:** Significant differences in dALFF values between RD patients and HCs at a sliding window size of 50TR.

Brain region	BA	Peak t-score	MNI coordinates (x, y, z)	Cluster size (voxels)
Temporal_Mid_R	21	3.8444	63, −3, −15	52
Frontal_Mid_L	–	4.7678	−24, 48, 9	249
Calcarine_R	–	4.1172	21, −96, 0	73
Putamen_L	–	3.7194	−18, 9, 0	58
Occipital_Mid_L	–	4.4051	−12, −105, 6	73
Frontal_Inf_Tri_R	–	3.5666	51, 30, 6	101
Parietal_Sup_R	5	−3.2846	24, −48, 69	69

dALFF, dynamic amplitude of low-frequency fluctuation; HCs, healthy controls; RD, retinal detachment; BA, Brodmann area; MNI, Montreal Neurological Institute; Temporal_Mid_R, right middle temporal gyrus; Frontal_Mid_L, left middle frontal gyrus; Calcarine_R, right calcarine; Putamen_L, left putamen; Occipital_Mid_L, left middle occipital gyrus; Frontal_Inf_Tri_R, right inferior frontal gyrus; Parietal_Sup_R, right superior parietal gyrus.

**Figure 3:**
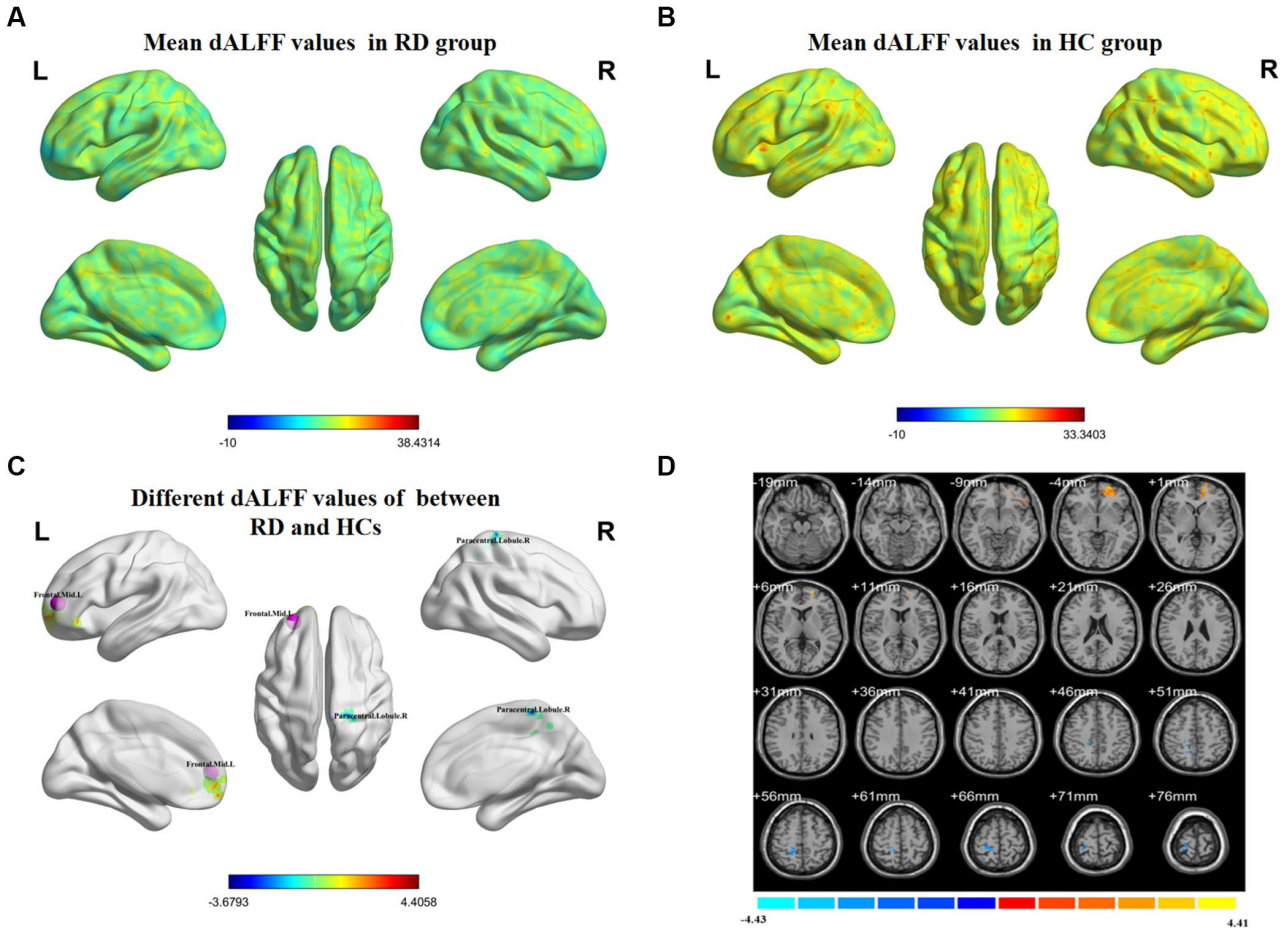
Spatial distributions of dALFF values in **(A)** RD patients (one-sample t-test) and **(B)** HCs (one-sample *t*-test). **(C,D)** Spatial distributions of dALFF values between RD patients and HCs (two-sample *t*-test). HCs, healthy controls; RD, retinal detachment; dALFF, dynamic amplitude of low-frequency fluctuation; L, left; R, right (two-tailed, voxel-level p < 0.05; GRF correction, cluster-level *p* < 0.05).

Figure 3 shows the spatial distribution of dALFF values between RD patients and HCs at a sliding window size of 80TR. In the left middle frontal gyrus, dALFF values were significantly higher in RD patients than in HCs. Conversely, in the right paracentral lobule, dALFF values were significantly lower in RD patients than in HCs (Figure 3; Table 4; two-tailed, voxel-level *p* < 0.05; GRF correction, cluster-level *p* < 0.05).

**Table 4:** Significant differences in dALFF values between RD patients and HCs at a sliding window size of 80TR.

Brain region	BA	Peak t-score	MNI coordinates (x, y, z)	Cluster size (voxels)
Frontal_Mid_L	–	4.4058	−27, 51, 9	133
Paracentral_Lobule_R	–	−3.6793	9, −36, 63	74

dALFF, dynamic amplitude of low-frequency fluctuation; HCs, healthy controls; RD, retinal detachment; BA, Brodmann area; MNI, Montreal Neurological Institute; Frontal_Mid_L, left middle frontal gyrus; Paracentral_Lobule_R, right paracentral lobule.

### 3.3. Clustered dALFF states

**Figure 4:**
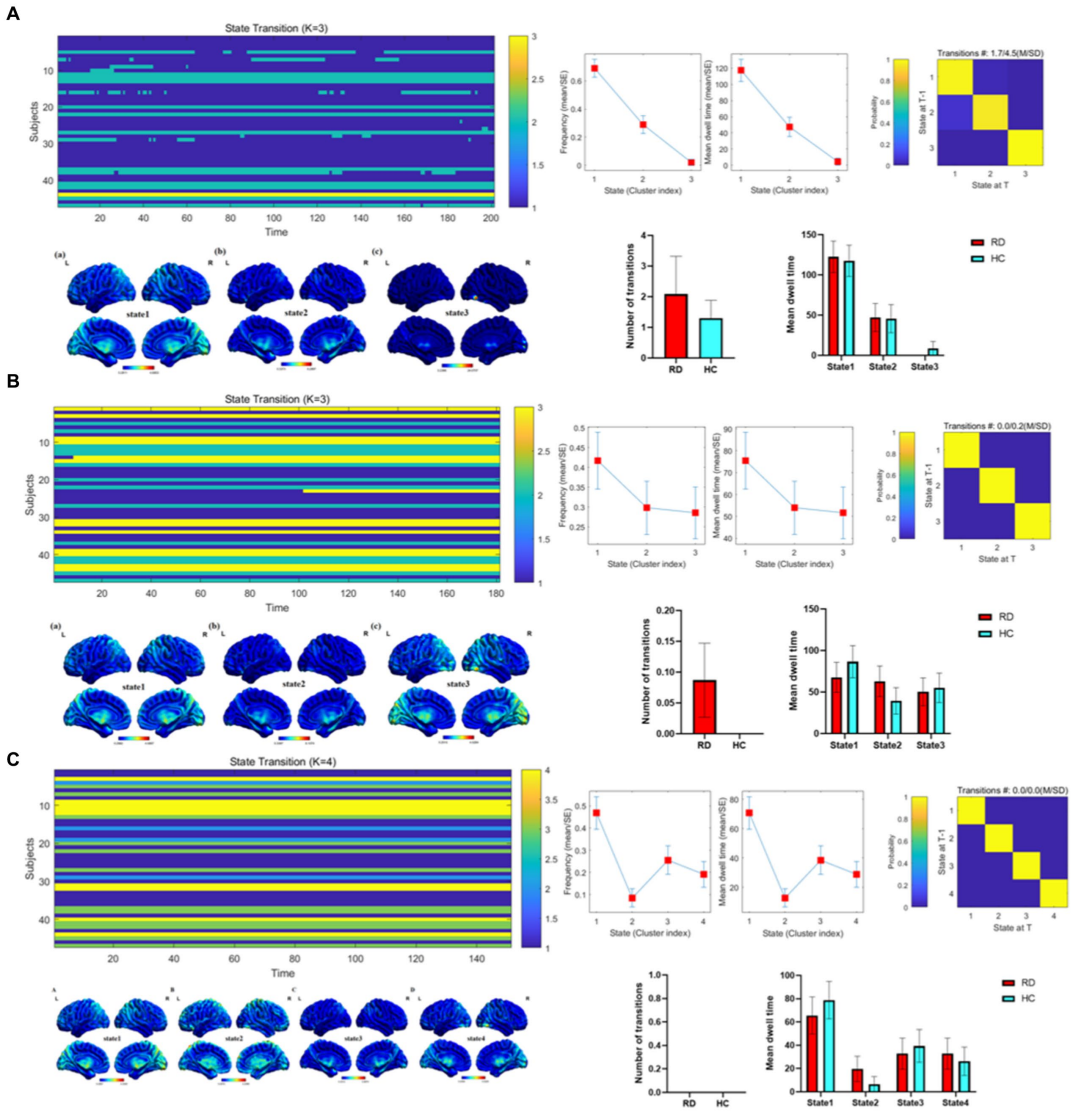
Temporal properties of dALFF patterns between RD and HC groups at sliding window sizes of 30TR **(A)**, 50TR **(B)**, and 80TR **(C)**.

Figure 4 depicts the overall state transition mode of all participants at sliding window sizes of 30TR, 50TR, and 80TR. The frequency and mean dwell time are shown for different states, along with the probability of transition between states.

### 3.4. Mean weighted dALFF values

**Figure 5:**
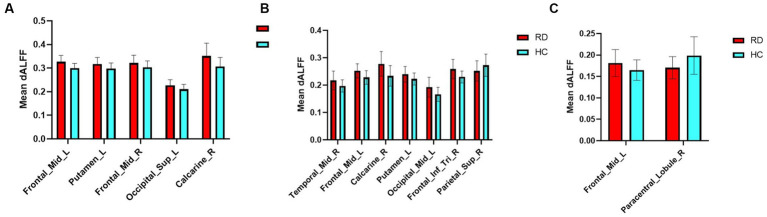
Mean weighted dALFF values of RD patients and HCs in altered brain regions at sliding window sizes of 30TR **(A)**, 50TR **(B)**, and 80TR **(C)**.

The mean altered dALFF values between RD patients and HCs at sliding window sizes of 30TR, 50TR, and 80TR are shown in Figure 5.

### 3.5. SVM classification results

**Figure 6:**
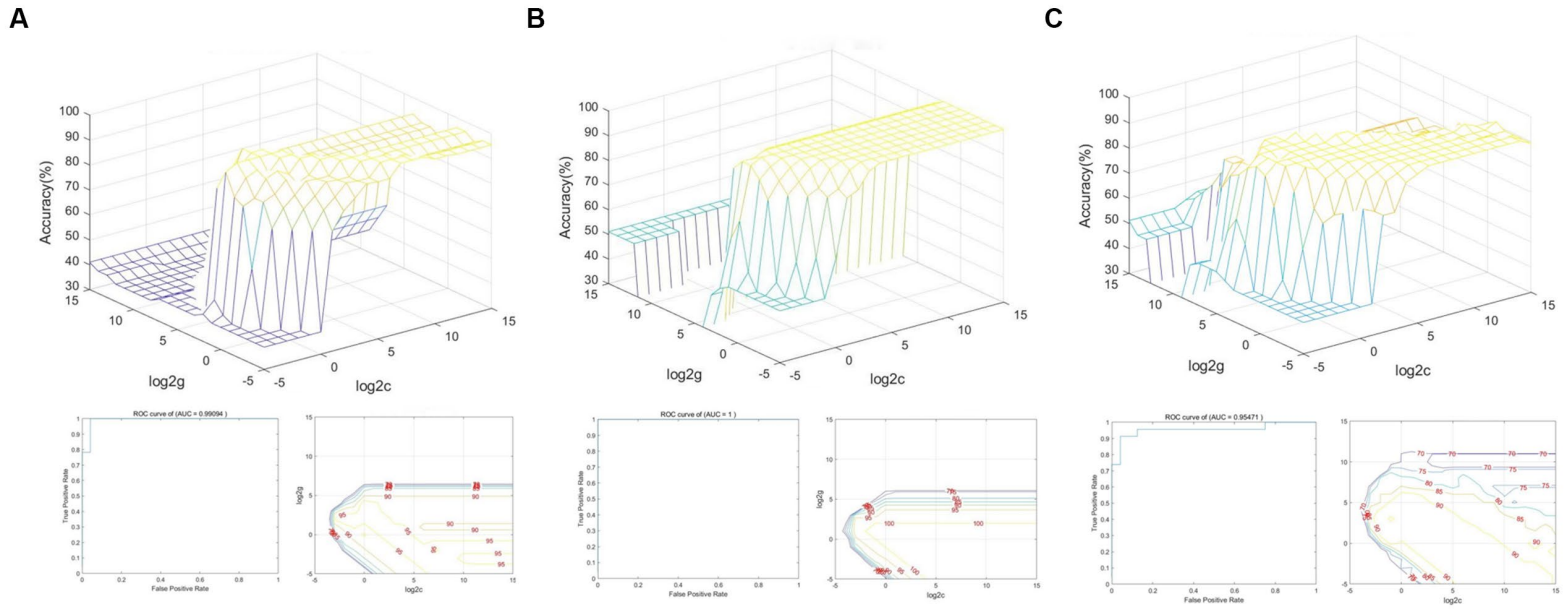
SVM classification of RD patients and HCs based on altered brain regions identified using sliding window sizes of 30TR **(A)**, 50TR **(B)**, and 80TR **(C)**.

Figure 6A shows that the total accuracy and area under the curve score of SVM classification, based on dALFF with a sliding window size of 30TR, were 97.87% and 0.99, respectively. Figure 6B shows that the total accuracy and area under the curve score of SVM classification, based on dALFF with a sliding window size of 50TR, were 100% and 1.00, respectively. Figure 6C shows that the total accuracy and area under the curve score of SVM classification, based on dALFF with a sliding window size of 80TR, were 93.62% and 0.95, respectively. These results indicate that the dALFF may be useful in the clinical diagnosis of RD.

### 3.6. Correlation analysis

Pearson and Spearman correlation analyses revealed that region of interest values in positive brain regions identified in RD patients at sliding window sizes of 30TR, 50TR, and 80TR were not correlated with HAMA scores (Table 5).

**Table 5:** Correlations between HAMA scores and brain regions with significant differences in RD patients at sliding window sizes of 30TR, 50TR, and 80TR.

Sliding window size	Brain region	Normality test (*p*-value)	Pearson correlation analysis (*p*-value)	Spearman correlation analysis (*p*-value)
30TR	Frontal_Mid_L	0.016	–	0.572
	Putamen_L	0.795	0.696	–
	Frontal_Mid_R	0.006	–	0.360
	Occipital_Sup_L	0.893	0.735	–
	Calcarine_R	0.084	0.821	–
50TR	Temporal_Mid_R	0.392	0.887	–
	Frontal_Mid_L	0.334	0.870	–
	Calcarine_R	0.258	0.673	–
	Putamen_L	0.898	0.961	–
	Occipital_Mid_L	0.020	–	0.683
	Frontal_Inf_Tri_R	0.009	–	0.734
	Parietal_Sup_R	0.185	0.407	–
80TR	Frontal_Mid_L	0.564	0.354	–
	Paracentral_Lobule_R	0.071	0.671	–

## 4. Discussion

This study showed that RD patients exhibited increased dALFF values in the bilateral middle frontal gyrus, left putamen, left superior occipital gyrus, left middle occipital gyrus, right calcarine, right middle temporal gyrus, and right inferior frontal gyrus. Additionally, RD patients showed significantly decreased dALFF values in the right superior parietal gyrus and right paracentral lobule (two-tailed, voxel-level *p* < 0.05; GRF correction, cluster-level *p* < 0.05). Using k-means clustering, three or four dALFF states were identified among all subjects. There were differences in state distribution and state properties between RD and HC groups. The number of transitions between the dALFF states was higher in the RD group than in the HC group. Based on dALFF values in various brain regions, the overall accuracies of SVM classification were 97.87, 100, and 93.62% under three different time windows; area under the curve values were 0.99, 1.00, and 0.95, respectively. No correlation was found between HAMA scores and regional dALFF.

### 4.1. Differences in dALFF variability

The frontal lobe, which constitutes approximately one-third of the cerebral cortex in humans, is a region of the brain that develops more slowly than other regions. Its wide-ranging and complex functions encompass nearly all cognitive neuropsychological activities. In this study, we found that the RD group had significantly higher dALFF values in the bilateral middle frontal gyrus and right inferior frontal gyrus, which are important for high-level cognition (e.g., executive function and working memory; Zheng et al., 2015). The decreased central vision, visual distortion, and reduced field of vision in RD patients may cause some motor execution impairment and poor memory. Kawashima et al. (2021) found that functional activation of the bilateral dorsolateral prefrontal cortex and middle frontal gyrus was reduced in Parkinson’s disease patients, presumably in relation to the pathophysiology of working memory disorder in such patients. Through voxel-based morphometry analysis, Zhao et al. (2021) demonstrated that gray matter volume was decreased in the bilateral middle frontal gyrus of patients with major depressive disorder, which may influence executive function in those patients. Chen et al. (2017) also revealed that FC between the right dorsolateral prefrontal cortex and right hippocampus was significantly reduced in long-term breast cancer survivors who had been treated with tamoxifen, implying significant defects in working memory and overall executive function. The above studies indicated that dALFF values in the bilateral middle frontal gyrus and right inferior frontal gyrus are significantly higher in RD patients than in HCs. Accordingly, we speculate that RD patients display motor execution impairment and poor working memory. In such patients, motor function and working memory are maintained before visual decline through compensatory mechanisms involving the bilateral middle frontal gyrus and right inferior frontal gyrus.

The putamen, a key component of the basal ganglia, is important for motor regulation (Romero et al., 2008; Vicente et al., 2012). We observed increased dALFF values in the left putamen of RD patients. Huang et al. (2018) found that the gray matter volume in the left putamen was increased among high myopia (HM) patients, suggesting that HM causes structural alterations in the bilateral putamen; this finding is consistent with the compensatory motor function observed in HM patients. Tong et al. (2021) revealed an increase in regional homogeneity in the left putamen among patients with iridocyclitis; they speculated that iridocyclitis causes functional changes in the putamen, which may lead to compensatory motor function. Hu et al. (2022) also demonstrated that voxel-based morphometry values were decreased in the left putamen of female menopausal dry eye patients, indicating that these patients may exhibit cognitive or motor impairments. Because RD causes sudden vision loss, patients cannot rapidly adapt to monocular vision, and their motor regulation is partially decreased relative to the pre-injury state; this phenomenon may also explain a portion of the increase in dALFF in the left putamen of RD patients. Therefore, we suspect that the increased dALFF in the left putamen compensates for the decrease in motor regulation among RD patients.

The occipital lobe, located in the posterior cerebral cortex, is responsible for visual perception. In this study, we found that dALFF values were increased in the left superior occipital gyrus, left middle occipital gyrus, and right calcarine in RD patients. Shao et al. (2021) reported increased FC density values in the left inferior occipital gyrus of RD patients, which may influence the brain’s effectiveness and accuracy in terms of processing visual digital information. Huang et al. (2017) also demonstrated that RD patients had decreased regional homogeneity in the right occipital lobe; they speculated that the decreased regional homogeneity reflected diminished synchrony among local brain regions, consistent with altered function in the primary visual cortex of RD patients. Wu et al. (2021) revealed that voxel-mirrored homotopic connectivity (VMHC) values in the bilateral calcarine were lower in bronchial asthma patients than in HCs. They suggested that the reduced VMHC values represent aberrant visual network function in asthma patients, leading to changes in visual function. The calcarine divides the occipital lobe into the cuneus above and lingual gyrus below; the primary visual cortex is located on both sides of the calcarine. Because the detached portion of the retina in RD patients cannot perceive light stimuli, the occipital lobe receives weaker visual signals, which may explain the increased dALFF values in the left superior occipital gyrus, left middle occipital gyrus, and right calcarine in RD patients. We suspect that these elevated dALFF values represent a compensatory mechanism by which the brain attempts to cope with vision loss in RD patients.

The temporal lobe, located below the lateral fissure, is divided into the superior temporal, middle temporal, and inferior temporal gyri; the inferior temporal gyrus is mainly involved in language comprehension (Dronkers et al., 2004). We found that dALFF values in the right middle temporal gyrus were increased in RD patients. Yu et al. (2008) reported that FC between the left superior temporal gyrus and middle temporal gyrus was decreased in patients with early blindness, whereas Huang et al. (2019) showed that degree centrality (DC) in the left inferior temporal gyrus was increased in patients with advanced monocular blindness. They speculated that the increase in DC compensated for vision loss in patients with advanced monocular blindness. Qi et al. (2022) showed that VMHC values in the bilateral medial temporal gyrus were decreased in patients with thyroid-associated ophthalmopathy, which may reflect diminished visual processing and attention in such patients. To our knowledge, there have been no reports of language comprehension problems among RD patients. We suspect that RD patients undergo a long period of visual improvement from the initial detachment until postoperative recovery; they may experience some reductions in the ability to learn and perceive external things, which could affect language comprehension. Thus, the elevated dALFF value in the right temporal gyrus may represent a compensatory mechanism for the decrease in language comprehension.

Finally, our study showed that dALFF values were decreased in the right superior parietal gyrus and right paracentral lobule in RD patients. The parietal gyrus, located above the medial parietal sulci, is involved in the transmission of visual information and the integration of visual movement (Caminiti et al., 1996; Iacoboni and Zaidel, 2004). Tan et al. (2018) found that the DC of the right superior parietal gyrus was decreased in comitant exotropia strabismus patients; this decreased DC may reflect functional impairment of the right superior parietal gyrus, which would explain eye movement dysfunction in such patients. In a previous study, we found that FC between the left V1 and L-SPG was increased in HM patients; we speculated that this increase in FC was a compensatory response to prevent impaired top-down control of visual attention in HM patients (Ji et al., 2023). Considering the previous findings, the decreased dALFF values in the right superior parietal gyrus of RD patients may reflect functional impairment in this brain area, which would explain why RD patients experience visual impairment. Moreover, the paracentral lobule extends from the lateral surface of the anterior and posterior central dorsal gyrus to the medial surface, which is closely associated with cognitive impairment (Mascalchi et al., 2014). Kim et al. (2019) found that changes in subnetworks, such as the paracentral lobule, were associated with cognitive scores in patients with subjective cognitive decline. However, Liang et al. (2020) showed that gray matter volume in the paracentral lobule was increased in patients with subjective cognitive decline; they suggested that the increased gray matter volume in the paracentral lobule represents a compensatory mechanism, although it is unclear whether the mechanism is associated with cognitive function. This notion is consistent with our findings that the dALFF value of the right paracentral lobule was decreased in RD patients, although we found no correlation between this brain region and HAMA scores. Therefore, we speculate that the decreased dALFF value of the right paracentral lobule in RD patients reflects inhibition of this brain region. However, there is no clear evidence of diminished cognitive function in RD patients.

In our study, the overall accuracies of SVM classification were 97.87, 100, and 93.62% under three different time windows; area under the curve values were 0.99, 1.00, and 0.95, respectively. Thus, dALFF may offer sensitive biomarkers for distinguishing patients with RD from HCs.

### 4.2. Differences in metrics of the dALFF states

Importantly, there were significant differences in the temporal characteristics of dALFF states between the two groups. Our results showed that the number of transitions in RD patients was higher than that in HC group at both 30TR and 50TR, while the number of transitions in RD patients and HC group was zero at 80TR. At the same time, compared with the HC group, RD patients at 30TR and 50TR showed 3 different time states, and RD patients at 80TR showed 4 different time states. In other words, during the entire resting state MRI scanning period, the brain’s working mode can be divided into three states at 30TR and 50TR, and the brain’s working mode can be divided into four states at 80TR. For patients with RD, state 1 accounted for a larger proportion of these states, suggesting that state 1 may represent a pattern of major brain activity in individuals with RD. In general, the mean dwell time and number of transitions are used as parameters in dynamic pattern analysis to describe state properties that represent brain functional activity and can be reconfigured during illness (Xu et al., 2023). Previous studies have found that patients with diabetic retinopathy exhibit three different temporal states, with state 1 occupying a larger proportion, while patients with diabetic retinopathy have a lower number of transitions than those in the HC group (Huang et al., 2021). Zhao et al. (2021) proposed that an increase in the number of transitions is associated with a decrease in the efficiency of information flow in brain networks. Therefore, we speculate that visual dysfunction leads to a decrease in the efficiency of information flow, which increases the number of conversions in patients with RD. This result also suggests that the whole brain integration of visual functions is abnormal.

## 5. Limitations

This study had some important limitations. First, it included a small number of RD patients. The lack of correlation between region of interest values in positive brain regions and HAMA scores may have been related to the small sample size. Second, the data were frequently affected by unavoidable factors in the fMRI environment (e.g., heartbeat, muscle beat, and respiratory motion).

## 6. Conclusion

In this study, we used the dALFF method, k-means clustering method and an SVM classification approach to explore dynamic changes in spontaneous brain activity among RD patients. Our findings offer important insights regarding the neuropathology that underlies RD and provide robust evidence that dALFF, a local indicator of brain activity, may be useful for clinical diagnosis.

## Data availability statement

The original contributions presented in the study are included in the article/supplementary material, further inquiries can be directed to the corresponding author.

## Ethics statement

The studies involving human participants were reviewed and approved by Medical Ethics Committee of the First Affiliated Hospital of Nanchang University. The patients/participants provided their written informed consent to participate in this study.

## Author contributions

YJ is responsible for writing manuscript. Y-yW is in charge of proofreading and refining the manuscript’s wording. QC, W-wF, S-qH, P-pZ, X-lC, B-lS, BW, and Q-yH contributed to data collection and statistical analyses. YJ and Y-yW designed the protocol and contributed to the MRI analysis. YJ, Y-yW, and X-rW designed the study, oversaw all clinical aspects of study conduct, and manuscript preparation. All authors contributed to the article and approved the submitted version.

## Funding

We acknowledge the assistance provided by the National Nature Science Foundation of China (grant no. 82160207), Key projects of Jiangxi Youth Science Fund (no. 20202ACBL216008), Science and Technology Plan of Jiangxi Provincial Health and Health Commission (202130156), and Postgraduate Innovation Special Fund Project in Jiangxi Province (YC2022—s198).

## Conflict of interest

The authors declare that the research was conducted in the absence of any commercial or financial relationships that could be construed as a potential conflict of interest.

## Publisher’s note

All claims expressed in this article are solely those of the authors and do not necessarily represent those of their affiliated organizations, or those of the publisher, the editors and the reviewers. Any product that may be evaluated in this article, or claim that may be made by its manufacturer, is not guaranteed or endorsed by the publisher.
